# Infarct Size Reduction in an Anterior ST-Elevation Myocardial Infarction Following “Optimized” Supersaturated Oxygen Therapy

**DOI:** 10.7759/cureus.53152

**Published:** 2024-01-29

**Authors:** Kamran Zaheer, Shaden Daloub, Mohamed Suliman, Kanaan Mansoor, Rameez Sayyed

**Affiliations:** 1 Internal Medicine, Marshall University Joan C. Edwards School of Medicine, Huntington, USA; 2 Cardiology, Marshall University Joan C. Edwards School of Medicine, Huntington, USA

**Keywords:** st-elevation myocardial infarction (stemi), supersaturated oxygen therapy (sso2), infarct size reduction, post-pci for lad stemi, primary percutaneous coronary intervention (pci)

## Abstract

This comprehensive case report documents the treatment of a 37-year-old female patient who presented with anterior ST-elevation myocardial infarction (STEMI). The patient underwent percutaneous coronary intervention (PCI), followed by an innovative therapy - optimized supersaturated oxygen therapy (SSO_2_). This therapy was chosen due to its potential to enhance myocardial salvage, particularly in severe MI cases like the patient. The report meticulously details the patient's clinical course, including the diagnostic procedures and the rationale behind opting for SSO_2_ therapy. It highlights the significant improvements observed post-therapy: enhanced left ventricular (LV) function and a remarkable reduction in the size of the LV apical aneurysm. These outcomes suggest a direct benefit of SSO_2_ in reducing myocardial damage. Finally, the report discusses the broader implications of these findings. It underscores the potential of optimized SSO_2_ therapy in clinical settings, particularly for patients with anterior MI. The case exemplifies how advanced therapeutic interventions like SSO_2_ can play a pivotal role in improving clinical outcomes post-MI, thereby advocating for its consideration in similar clinical scenarios.

## Introduction

For nearly three decades, the gold standard treatment for acute myocardial infarction (AMI) has been urgent revascularization, also known as primary percutaneous coronary intervention (PCI) [1]. When compared to conservative therapy, which includes thrombolysis, it reduces mortality and enhances cardiovascular outcomes [2]. One important factor influencing hospitalization for heart failure and one-year all-cause mortality is infarct size. In primary PCI, optimizing myocardial salvage and decreasing infarct size to minimize post-MI sequelae of heart failure and mortality continues to be a crucial objective [1,2]. Despite high rates of epicardial coronary flow restoration by PCI, myocardial salvage is frequently suboptimal in ST-elevation myocardial infarction (STEMI), even though early reperfusion therapy has decreased mortality over the past few decades [3]. Numerous mechanisms, including reperfusion injury, microcirculatory dysfunction or no-reflow, and late reperfusion, have been linked to this [4].

Supersaturated oxygen therapy (SSO_2_) infused into the left anterior descending coronary artery (LAD) was used to treat patients with anterior STEMI in the pivotal Acute Myocardial Infarction With Hyperoxemic Therapy II (AMIHOT II) trial. The results showed a significant reduction in infarct size at 14 days when compared with the control group [5]. However, because the SSO_2_ group required larger or more femoral arterial sheaths, hemorrhagic complications were more common. In addition, nonsignificant trends were observed for increased stent thrombosis and death at 30 days, which may have been caused by the SSO_2_ being delivered through an indwelling catheter in the stented area of the LAD [6].

This led to a modification of the intracoronary SSO_2_ delivery technique, resulting in "optimized" SSO_2_ delivery whereby, after PCI, hyperoxemic blood was infused into the left main coronary artery (LMCA) ostium via a diagnostic catheter. One of the systems that can provide optimized SSO_2_ therapy is ZOLL TherOx (ZOLL Medical Corporation, USA). We are presenting the case of a 37-year-old female patient who received SSO_2_ therapy following PCI for LAD STEMI.

## Case presentation

**Figure 1:**
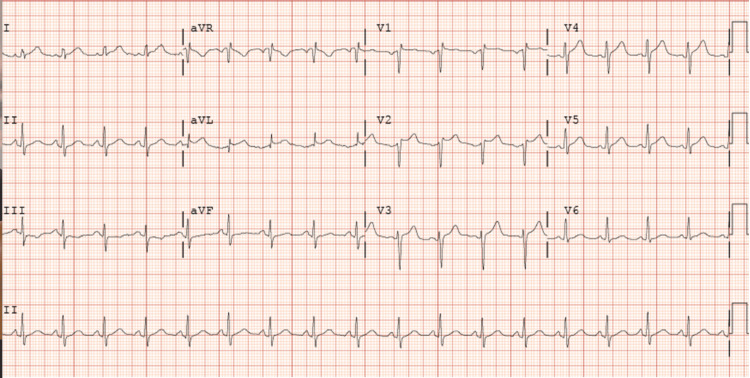
EKG showed ST elevation in leads V2, V3, and V4 correlating with anterior STEMI with reciprocal changes in the inferior leads III and aVF. EKG: electrocardiogram, STEMI: ST-elevation myocardial infarction, aVF: arteriovenous fistula

A 37-year-old female with a past medical history significant for nicotine dependence and uncontrolled diabetes presented to the emergency room with the chief complaint of chest pain. She described her chest pain as constant, severe, retrosternal, and radiating to her neck, which started around 40 minutes before presenting to the ER with no apparent aggravating or relieving factors. The pain was also associated with shortness of breath, nausea, and vomiting. In the emergency department, she was found to have dynamic EKG changes concerning for anterior STEMI (Figure 1).

**Figure 2:**
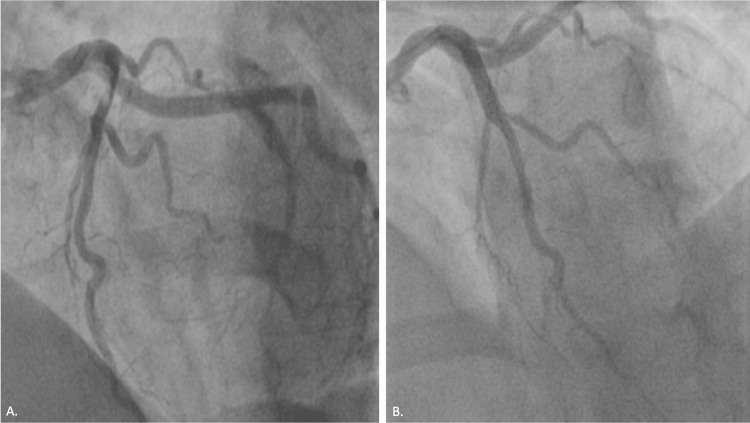
(A) A thrombus in her left anterior descending artery. (B) Same lesion after PTCA, primary PCI, and aspiration thrombectomy to the left anterior descending artery. PTCA: percutaneous transluminal coronary angioplasty, PCI: percutaneous coronary intervention

The patient was taken emergently to the cath lab. Catheterization was done after around 67 minutes from the onset of chest pain and revealed a thrombus in her LAD artery along with left ventricular (LV) dysfunction (Figure 2A). After reviewing the angiographic data, we proceeded with percutaneous transluminal coronary angioplasty (PTCA), primary PCI, and aspiration thrombectomy to the LAD artery (Figure 2B).

**Figure 3:**
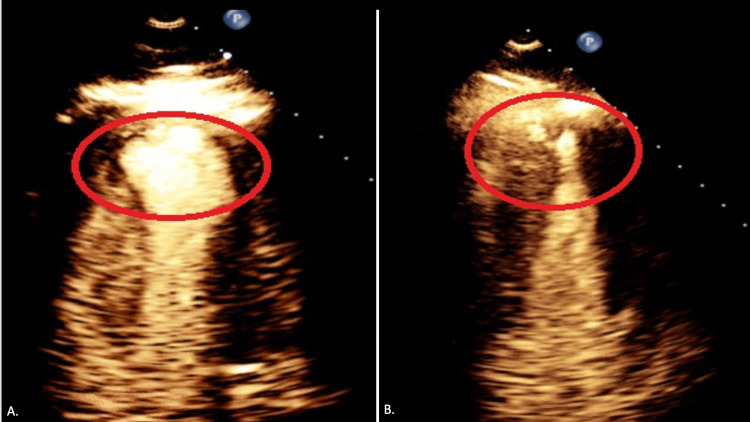
A. Aneurysmal dilation of the LV apex post-procedure (red oval). B. LV aneurysm that became smaller at one-month follow-up (red oval). LV: left ventricular

After successfully treating the culprit lesion, we decided to proceed with SSO_2_ TherOx treatment for anterior STEMI. Right femoral access was obtained, and a 6 French sheath was placed. We then used the JL 4.0 catheter and engaged the left coronary system. The sheath was sutured in place. The TherOx system blood draw line was connected to the sheath, and re-infusion was done from the JL 4.0 catheter that was left in place to infuse supersaturated oxygen into the left coronary system for a total of 60 minutes. Initial EKG revealed hypokinetic apical and apical septal LV segment. There was a 3 cm-wide apical ballooning (Figure 3A). The LV ejection fraction was estimated to be 50%. The rest of her hospital stay was uneventful. The patient was started on goal-directed medical therapy for coronary artery disease. The patient was discharged with a one-month follow-up from the hospital.

On follow-up, the patient did not have any complaints. She had a repeat EKG, which showed that she only had a small remnant LV ballooning (Figure 3B). It measured 1.8 cm wide, a biplane LV ejection fraction of 59.8%, and recovery of the apical septal hypokinesia.

## Discussion

Several pharmacologic techniques, such as intracoronary adenosine, nitroprusside, and abciximab infusions [7], have been used in the past to enhance microcirculatory function, avoid reperfusion injury, and decrease infarct size in patients with STEMI [8]. Nevertheless, there is no evidence that any of these therapies enhance clinical results. In patients with large anterior STEMI, the intracoronary delivery of SSO_2_ significantly decreased infarct size following primary PCI, a parameter strongly predictive of subsequent death and heart failure rehospitalizations [9]. Thus, SSO_2_ became the first medication to be shown to improve myocardial salvage and decrease infarct size in a pivotal, suitably powered, randomized trial [5,6,9].

SSO_2_ therapy proved safe and feasible for treating acute anterior STEMI in humans undergoing primary PCI. Larger clinical trials that can directly show a reduction in major adverse cardiovascular events are still lacking, but data from nearly 360 patients suggest that treating anterior STEMI within six hours of the onset of symptoms may be able to reduce infarct size. According to aggregate data from AMIHOT I, AMIHOT II, and IC-HOT, SSO_2_ therapy may help the infarct size of those patients decrease from roughly 25% to 27% of the LV to roughly 19% [5,6,10].

Although the primary composite safety endpoint of the AMIHOT II trial was met, bleeding complications were more common. In patients treated with SSO_2_, there were indications of increased 30-day rates of myocardial rupture, stent thrombosis, and mortality [9]. In the IC-HOT study, SSO_2_ delivery was "optimized" to be selectively infused to the origin of the left main coronary artery instead of the LAD at the stent site due to these concerns [11]. The IC-HOT study was an open-label, single-arm, prospective investigation. This instance demonstrates how SSO_2_ therapy helps patients. In the beginning, the patient had a sizable LV ballooning and mild LV dysfunction. Subsequent surveillance echocardiography, however, revealed improved LV function and a 1.8 cm versus 3.0 cm reduction in the size of the LV apical ballooning. In light of the results of this case, we recommend SSO_2_ therapy for patients suffering from LAD STEMI.

It will be interesting to observe in the future if the patient benefits more from a combination of different strategies for reducing infarct size in addition to primary PCI. However, for this to work, each of the individual interventions must be beneficial on its own. Appropriate clinical trials are either in the planning stages or currently underway [12].

## Conclusions

In addition to primary PCI, SSO_2_ therapy is a newly available treatment option for patients with anterior STEMI. The IC-HOT trial was the only one used to assess the effectiveness of the "optimized" SSO_2_ therapy; however, it was constrained by its single-arm, open-ended design. Therefore, additional clinical research is required to examine the practical effects of "optimized" SSO_2_ therapy. In this instance, we offer a very positive result for a patient who received "optimized SSO_2_ therapy."
